# Interactions between stretch and startle reflexes produce task-appropriate rapid postural reactions

**DOI:** 10.3389/fnint.2015.00002

**Published:** 2015-01-28

**Authors:** Jonathan Shemmell

**Affiliations:** Sport and Exercise Sciences, Brain Health Research Centre and School of Physical Education, University of OtagoDunedin, New Zealand

**Keywords:** long-latency stretch reflex, startle response, startReact, movement preparation, motor cortex

## Abstract

Neural pathways underpinning startle reflex and limb stretch reflexes evolved independently and have served vastly different purposes. In their most basic form, the pathways responsible for these reflex responses are relatively simple processing units that produce a motoric response that is proportional to the stimulus received. It is becoming clear however, that rapid responses to external stimuli produced by human and non-human primates are context-dependent in a manner similar to voluntary movements. This mini review discusses the nature of startle and stretch reflex interactions in human and non-human primates and the involvement of the primary motor cortex in their regulation.

Like all animals, humans have inherited most of our anatomical structures and physiological functions from our ancestors. Some of these we have retained largely intact and some we have modified from their original form to suit our own purposes. We have also added some of own structures and functions that supersede or regulate those we have inherited. The purpose of this review is to discuss the contribution of stretch reflex and startle response circuits to the flexibility of rapid postural responses in humans and the possibility that rapid, goal-directed actions may be subserved by interactions between separate cortical and subcortical neural circuits. The focus of the review is on the generation, rather than the context-dependent modulation of these rapid responses.

I will begin by defining rapid postural responses as being muscular contractions that occur in response to an external perturbation, detected by one or more sensory modalities, and with a latency that is shorter than the most rapid voluntary response. This definition immediately raises the problem of how we determine that an action is voluntary and, although it is a somewhat unsatisfying resolution, I will define the most rapid voluntary actions as those that result from a person being asked to make a predefined response as rapidly as possible to a detectable, but not startling, stimulus. The magnitude of the stimulus becomes critical in any discussion of startle responses and we therefore arrive at a relative definition of voluntary actions that will suffice for the purposes of this review without being ideal. As I will discuss, distinctions between voluntary and involuntary responses are becoming blurred by discoveries about the flexibility of “involuntary” actions, and attempts to distinguish them may be futile.

## Evolutionary functions of rapid motor reactions

Rapid, “involuntary” postural responses are not unique to humans. This type of motor action can be observed in early chordates, such as lampreys (Currie and Carlsen, 1985), with simple central nervous systems animals and even in animals with no central nervous system, such as jellyfish and other Cnidaria (Mackie, 1984). In these types of animals, the expression of rapid postural responses to startling stimuli often takes the form of an escape reflex to avoid predators. While the goal of these escape responses is consistent across a wide variety of animals, the pattern of muscle activation required to facilitate escape necessarily differs between animals with different body morphologies. In short-bodied aquatic animals for example, the escape response is characterized by all-or-none contraction of the muscles on one side of the body, resulting in the assumption of a C-type posture before contractions on the opposite side of the body produce the tail flick required for a rapid getaway (Eaton et al., 1977). In contrast, escape in long-bodied teleosts is enabled by co-contraction of muscles on both sides of the body, allowing rapid withdrawal of the head as the animal assumes a flattened S-shape posture (Rock, 1980). The pattern of muscle contraction required for this type of head withdrawal changes with the posture of the animal, and it has been shown that even larval lamprey have the capacity to modify the pattern of muscle activity of the escape response to compensate for changes in their initial posture (Currie and Carlsen, 1985). Escape responses in teleost fish, and presumably most animals, are also modified to account for differences in the location of the threatening stimulus (Eaton and Emberley, 1991). The flexibility of the escape response in even animals with simple nervous systems demonstrates the important role of multisensory feedback for online adaptation of rapid postural and escape responses.

Given the need for rapid predator avoidance in animals with vastly different body types, the expression of these responses has been necessarily altered to suit the sensorimotor requirements of each anatomical form. Hale et al. (2002) have demonstrated that the expression of escape behavior is not conserved even across four species of fish with similar anatomy. The neural circuits mediating escape responses also appear to have undergone evolutionary adaptation, although perhaps less so, as several characteristics of the circuit have remained invariant. The need for rapid conduction from the sensory organs that detect a threat to the peripheral musculature that initiates escape appears to have constrained the type of neurons involved in the escape circuit to have axons of particularly large diameter [described as “giant fibers” in many animals] (Curtis and Cole, 1942; Mulloney, 1970; Eaton et al., 1977; Lingenhöhl and Friauf, 1992). These fast-conducting cells are invariably located within the brainstem or homologous hindbrain structure and often receive input from multiple sensory modalities including the auditory, visual and somatosensory systems. Even in mammals, who lack axons sufficiently large to be labeled as “giant,” there exist populations of cells within the ventrocaudal part of the nucleus reticularis pontis caudalis with large soma and direct projections to the spinal cord (Mitani et al., 1988) that have been implicated in rapid responses to startling stimuli (startle reflexes) (Krasne and Edwards, 2002; Cluff and Scott, 2013; Safavynia and Ting, 2013).

The stimulus-driven reflexes initiated by giant brainstem cells can be suppressed or facilitated in a number of animals according to moment-to-moment functional requirements. An example of this type of reflex regulation can be found in the squid, which acquires the capacity for startle reflex inhibition during development to allow for the capture and consumption of small, fast-moving prey (copepods) (Preuss and Gilly, 2000). If squid do not encounter copepods in their environment during early development they do not acquire the ability to suppress the startle reflex. Similar types of startle reflex regulation, both inhibition and facilitation, are demonstrated by crayfish (Krasne and Edwards, 2002) and rats (Prosser and Hunter, 1936). The function of this startle reflex pathway has also been observed to change over an evolutionary time scale in Tahitian moths that have evolved in the absence of bats (Fullard et al., 2004). The startle reflex in these moths has been modified such that responses to high frequency auditory stimuli (similar to bat echolocation sounds) have been substantially reduced, although neural circuit underlying the startle reflex has been retained (Fullard et al., 2007). These examples demonstrate the potential for startle reflexes to be regulated rapidly and reversibly as well as very slowly but more permanently in animals with relatively simple nervous systems compared to the human nervous system. It is unsurprising then that the expression of rapid postural reactions in humans appears to be regulated in a manner that compensates for many postural and environmental factors.

## Contributions of stretch reflexes to rapid motor reactions in humans

Humans are capable of producing rapid responses to large somatosensory, auditory or visual stimuli. In the upper limb, muscular responses to rapid stretch are detectable as early as 20 ms after the stretch begins, this response is referred to as the short-latency or myotatic stretch reflex (Liddell and Sherrington, 1924). If the muscle stretch lasts longer than 35 ms (±5.5 ms), a second muscle contraction can be observed, beginning 50–60 ms after the onset of muscle stretch (Lewis et al., 2005), referred to as the long-latency or transcortical stretch reflex. Both of these responses are assumed to be involuntary because the fastest voluntary muscular responses to smaller stimuli of the same type are initiated 90–100 ms after the stimulus (Hammond, 1955). In contrast to the short-latency stretch reflex, which is subserved entirely by cells within the spinal cord and peripheral nervous system, the long-latency stretch reflex appears to involve the primary motor area of the cortex (Matthews, 1991; Shemmell et al., 2009; Pruszynski et al., 2011) as well as other brain regions including the cerebellum (Vilis et al., 1976; Strick, 1983). The role played by the stretch reflex in postural maintenance and movement is not yet clear and has been the subject of debate in the scientific community for decades. Originally believed to be a mechanism by which postural perturbations were corrected, it has since been demonstrated that the muscle contractions produced by the stretch reflex are insufficient for this role (Crago et al., 1976). On this basis it has been argued that the purpose of the stretch reflex is to regulate muscle stiffness, and therefore limb impedance (Sinkjaer and Hayashi, 1989; Carter et al., 1990; Kearney et al., 1997). Some of the factors influencing the expression of the long-latency stretch reflex however, suggest that either its role is more complicated or that there are multiple mechanisms for postural regulation being invoked. Some of the major factors influencing the long-latency stretch reflex are outline below.

### Stability of the limb and environment

Cortical regulation of the long-latency stretch reflex circuit appears to imbue this response with the capacity for subtle, task-appropriate modulation and coordination. For example, the amplitude of the long-latency stretch reflex is larger during interactions with compliant devices than those offering high levels of stiffness (Doemges and Rack, 1992; Dietz et al., 1994; Perreault et al., 2008). When environmental instability is greater in some directions than others, the amplitude of the long-latency stretch reflex in many muscles is greatest when the direction of greatest environmental instability aligns with the direction of greatest limb instability (Krutky et al., 2010). This reflex modulation allows changes in arm stiffness to rapidly compensate for instabilities in the environment (Kimura et al., 2006; Franklin et al., 2007; Wagner and Smith, 2008; Ahmadi-Pajouh et al., 2012; Cluff and Scott, 2013). That is, the long-latency stretch reflex appears to be regulated according to the levels of stability simultaneously offered by the environment with which one interacts and the configuration of the limb with which those interactions are made.

### Body and limb posture

Long latency stretch reflexes in soleus and tibialis anterior muscles have also been shown to be sensitive to changes in body posture, being substantially greater during standing than when lying supine despite equivalent levels of muscle activation in both conditions (Nakazawa et al., 2003). The long latency postural response in the upper limb also appears to be altered depending upon the amplitude and direction of postural perturbations at limb segments distal to the recorded muscle (Kurtzer et al., 2008), demonstrating that rapid postural responses are distributed to muscles that have not been stretched or otherwise stimulated and that expression of the long latency response reflects an understanding of limb mechanics.

### Imminent goal-directed movements

The long latency postural response, at least in the upper limbs, is also modulated according to the voluntary goal of an upcoming action (Pruszynski et al., 2008; Crevecoeur et al., 2013). Hammond was the first to demonstrate that perturbations of elbow posture could induce a response in the biceps brachii 50–60 ms after the onset of the perturbation (Hammond, 1955) that was alterable according to the intention of the participant to assist or resist the perturbation (Hammond, 1956). Evarts and colleagues were also able to demonstrate that pyramidal tract neurons originating in the motor cortex become active well in advance of such responses and that the firing rate of individual neurons was associated with the direction of the intended movement (Evarts and Tanji, 1974; Tanji and Evarts, 1976). It has since been shown that rapid perturbations of limb posture can hasten whole patterns of muscle activity that are appropriate for an intended movement, beginning at the time associated with the long-latency stretch reflex but also involving non-stretched muscles (Koshland and Hasan, 2000). Similarly, task-specific patterns of muscle activity have been observed in response to limb perturbations when the direction of the required response is unpredictable prior to the perturbation (Pruszynski et al., 2011; Omrani et al., 2014). The release of task-specific patterns of muscle activity has led to confusion as to whether the long-latency stretch reflex is a regulator of limb impedance to minimize postural disturbances (reaction) or whether it also plays a role in movement planning (action).

Some investigators have suggested that some of the confusion about the role of the long-latency stretch reflex may be caused by the superposition of two reflexive responses, one regulating limb impedance and one involved in the preparation and release of motor plans. Based initially on similarities in the timing of the long-latency stretch reflex and the startle response in many muscles, it has been suggested that activation of the startle response circuit may be responsible for releasing planned motor actions (Valls-Solé et al., 1999; Rothwell et al., 2002).

## Contributions of startle responses to rapid motor reactions in humans

For many animals, the neural circuit underlying responses to startling or immediately threatening stimuli involves connections from a number of sensory receptor systems onto large, rapidly conducting neurons in the brainstem (Mittenthal and Wine, 1978; Ritzmann and Camhi, 1978; Koto et al., 1981). This also appears to be true for humans, with the nucleus reticularis pontis caudalis identified by anatomical and electrophysiological studies as the most likely site at which auditory, vestibular and somatosensory stimuli summate to trigger the motor portion of the startle response (Davis et al., 1982; Lingenhöhl and Friauf, 1992; Yeomans and Frankland, 1995; Yeomans et al., 2002). In humans, triggering the startle response at rest with a loud auditory stimulus produces activity in many muscles throughout the body, almost always including both the orbicularis oculi (OO) and sternocleidomastoid (SCM) and with predominant flexor activity in limb muscles (Landis and Hunt, 1939). This pattern of muscle activity is altered radically when a startling auditory stimulus is applied before, or coincident with, a prepared voluntary action (Valls-Solé et al., 1999; MacKinnon et al., 2007; Ravichandran et al., 2013). When an action has been prepared, the pattern of muscle activity evoked by a loud auditory stimulus closely resembles that of the prepared movement, while also often involving activation of the OO and SCM muscles (Valls-Solé et al., 1999; Ravichandran et al., 2013). The major difference between prepared actions that are triggered by innocuous or startling auditory stimuli is the latency of the response, with startling auditory stimuli triggering the initiation of prepared forearm actions around 100 ms earlier than low volume stimuli (Valls-Solé et al., 1999). A similar hastening of prepared actions, with associated OO and SCM activity, has also been observed following rapid joint perturbations (Ravichandran et al., 2013) and whole-body postural perturbations (Campbell et al., 2013; Safavynia and Ting, 2013), confirming that rapid goal-directed responses: (i) are triggered by the same sensory modalities as startle responses, (ii) are initiated at the same time in limb muscles as startle responses and (iii) activate the muscles essentially involved in startle response expression (OO and SCM). This is consistent with the idea that auditory, vestibular and somatosensory inputs to the reticularis pontis caudalis are capable of activating startle circuitry and triggering rapid postural responses that resemble their voluntary counterparts. The electromyographic evidence accumulated to date therefore supports the involvement of the neural circuit underlying the startle reflex in the rapid expression of flexible postural responses to startling stimuli. This response has been termed the startReact response. An important caveat to this however, are observations that postural adjustments made prior to stepping can also be released early by non-startling stimuli (Delval et al., 2012), the likelihood of early release being related to the strength of the stimulus. This suggests a system of (at least) two response pathways in which the faster pathway is more likely to be engaged as stimulus strength increases.

Given similarities in the timing of long-latency stretch reflex and startReact expression in many muscles (long latency stretch reflex onset ~57 ms, Lewis et al., 2005 and startReact onset ~73 ms, Ravichandran et al., 2013), temporal overlap of the two reflexes could explain the wide variety of conditions under which the magnitude of “involuntary” postural responses is observed to change. While the evidence for temporal overlap of two “involuntary” responses is now strong following auditory, somatosensory and vestibular stimuli (Alibiglou and MacKinnon, 2012; Nonnekes et al., 2014), the neural basis for this type of superposition remains a source of debate. A number of investigators have suggested that cortically-initiated preparation for action alters the state of the startle circuit in a manner that results in the full expression of a planned action when the startle circuit is subsequently activated (Rothwell et al., 2002; Shemmell et al., 2009). An anatomical substrate for this type of cortical regulation of startle circuits exists in the form of dense disynaptic connections from the primary motor cortex to the reticularis pontis caudalis via the zona incerta (Shammah-Lagnado et al., 1987). Tanji and Evarts have also provided evidence that pyramidal tract neurons within the primary motor cortex change their firing rate during movement preparation as soon as an upcoming movement is identified (Tanji and Evarts, 1976), providing a signal capable of altering startle circuit excitability. Unfortunately, a definitive investigation of links between motor cortex activity and that of cells in areas of the reticular formation that have been implicated in the startle circuit has not yet been carried out during movement preparation. The effects of transient primary motor cortex inhibition however, have provided some interesting insights into interactions between cortical and brainstem centers involved in startle and startReact responses.

Inhibiting the primary motor cortex with transcranial magnetic stimulation (TMS) during the period in which a startReact response would be observed has produced varying effects on muscle activity. In some studies, startReact responses are clearly delayed by the cortical stimulus (Alibiglou and MacKinnon, 2012; Stevenson et al., 2014), suggesting a critical involvement of the motor cortex in the generation of the startReact response. Several researchers have proposed models for the circuits underlying startle and startReact responses (Carlsen et al., 2011, 2012; Alibiglou and MacKinnon, 2012) in which startling auditory stimuli are transmitted to the primary motor cortex where they trigger the activation of cells that are biased toward producing a prepared movement. The proposed model describes separate pathways for startle and startReact responses, with the latter being dependent upon the same cortical output neurons as voluntary commands. This model fits previous accounts of preparation-dependent activity of motor cortical output neurons (Tanji and Evarts, 1976) and accounts for similarities in preparation-dependent modulation of long latency stretch reflexes and startReact responses (Kimura et al., 2006; Pruszynski et al., 2011; Spieser et al., 2013; Stevenson et al., 2014). The reliance of startReact responses on a transcortical pathway may also explain observations that prepared actions can be triggered early by non-startling stimuli (Delval et al., 2012). There have been at least two observations, however, of prepared actions being released within a period of cortical inhibition sufficiently powerful to suppress all voluntary activity in a target muscle (Shemmell et al., 2009; Spieser et al., 2013), a combination of events that would not be possible if startReact responses and voluntary motor actions were dependent on the same set of corticospinal tract neurons. These observations are more consistent with models of startReact that emphasize the contribution of subcortical structures (Valls-Solé et al., 2008).

**Figure 1:**
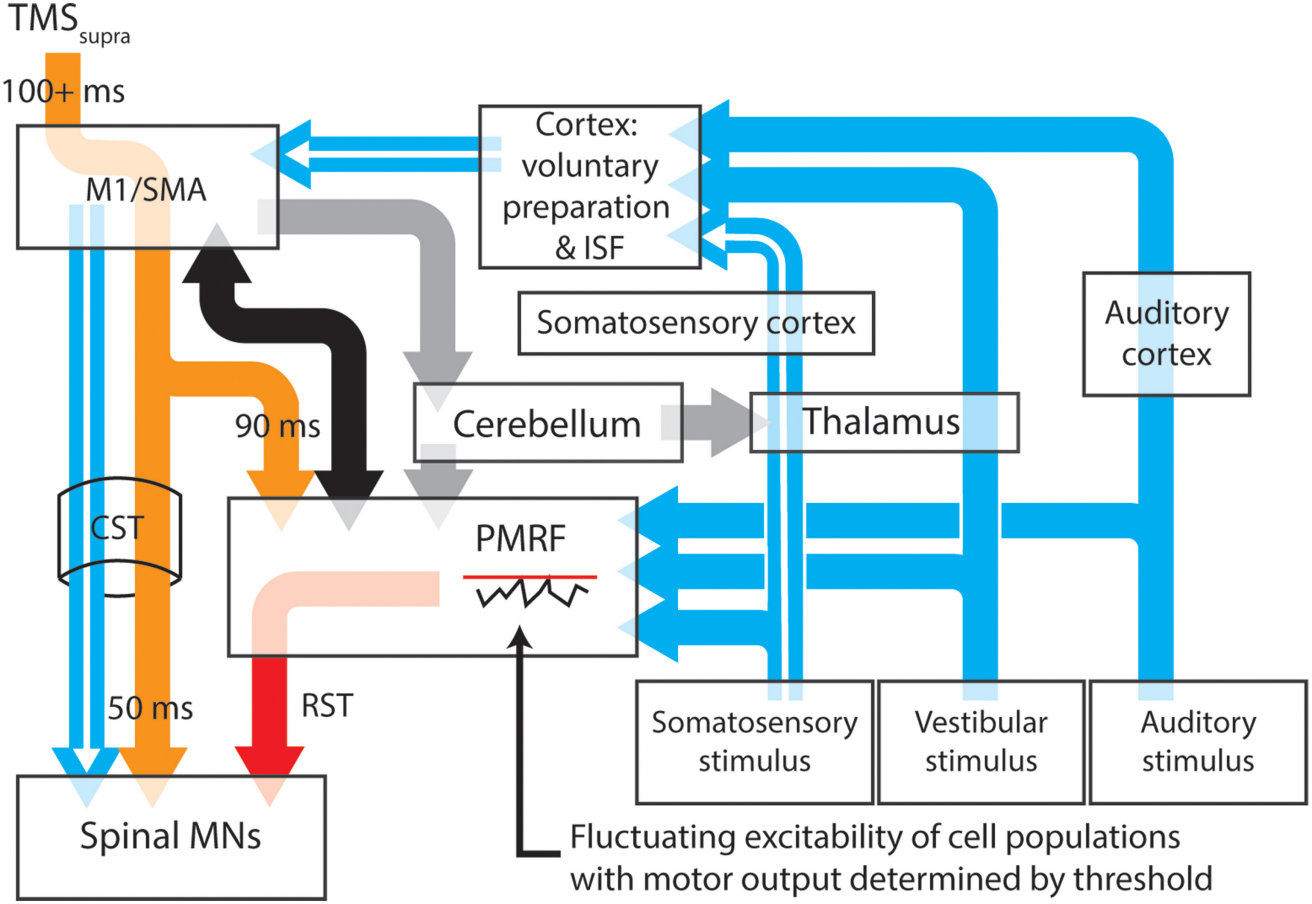
**A proposed model for the neural pathways subserving the expression of long latency stretch reflexes, startle and startReact responses**. Transcortical contributions to long latency stretch reflexes are enabled by a pathway that involves the primary somatosensory and motor cortices before descending to muscles through the corticospinal tract (white arrows). Stimulation of somatosensory, vestibular or auditory systems results in transmission of these signals to the cortex and the pontomedullary reticular formation (blue arrows), each of which have thresholds for activating output cells. Inhibition of the primary motor cortex (or areas involved in movement preparation) by application of suprathreshold TMS (orange arrows) results in almost simultaneous inhibition of cells within the spinal cord and PMRF, although for different periods of time. Spinal motoneurons appear to be inhibitied for ~50 ms after TMS, while cells within the PMRF are inhibited for ~90 ms in non-human primates. Output cells within the motor cortex however, can be inhibited for up to 200 ms (Strick, 1983). During periods of cortical inhibition, activation of the corticospinal tract is not possible, although a combination of cortico-reticulospinal input (black arrows) and sufficiently large sensory input can still activate reticulospinal tract cells (red arrow) after any TMS-induced inhibition of PMRF ceases. The likelihood of PMRF output (startle or startReact responses) is determined in this model by the magnitude of the sensory input and the instantaneous excitability of PMRF cells. A similar situation is likely to exist at the cortical level (see Alibiglou and MacKinnon, 2012). Task, posture and stability-dependent regulation of stretch reflex and startReact responses likely involves input from the cerebellum to both the primary motor cortex and reticular formation (gray arrows). In this figure thalamocortical projections to regions responsible for voluntary motor preparation, but projections directly to M1 may also play an important role in modulating rapid responses. Output nuclei and detailed information about synapse locations for each pathway have been omitted from this figure. M1, Primary motor cortex; SMA, supplementary motor area; PMRF, pontomedullary reticular formation; ISF, intersensory facilitation; MN, motoneuron.

It may be possible to account for the release of prepared actions within a period of cortical inhibition with a model that attributes the release of these actions to startle circuitry in the brainstem (Figure 1). The proposed model has the benefit of removing the separation between startle and startReact responses, instead explaining both responses as the result of subcortical “startle” circuits, explaining why cortical inhibition longer than 100 ms (Shemmell et al., 2009; Spieser et al., 2013), stroke (Honeycutt et al., 2015) or pathological degeneration of the corticospinal tract (Nonnekes et al., 2014) do not eliminate startReact expression. This model includes well-described transcortical sensorimotor pathways that provide the capacity for transcortical reflex transmission and the generation of longer latency task-specific actions. Factors such as the magnitude of the sensory stimulus and the predictability of the required response may determine the likelihood of motor actions being initiated within the reticular formation.

The delaying effect of TMS on startReact responses is accounted for in the model with evidence that TMS of motor cortical cells also has inhibiting effects on cells within the reticular formation. A period of prolonged inhibition (up to ~90 ms following a stimulus) of reticular formation neurons has been described by Fisher et al. (2012) following TMS of the primary motor cortex in monkeys. Interestingly, experiments in which startReact responses are preceded by TMS (Shemmell et al., 2009; Alibiglou and MacKinnon, 2012; Spieser et al., 2013; Stevenson et al., 2014) show the startReact response initiated ~100 ms after the TMS pulse, shortly after the putative release of inhibition within the reticular formation. The TMS-induced inhibition within the reticular formation would presumably be limited to neurons influencing the same muscles as the stimulated cortical neurons, explaining why activation of muscles innervated by facial nerves (SCM and OO) is not delayed by stimulation within the motor cortex representation of the upper limb (Stevenson et al., 2014). Further support for the idea that startReact responses are released through reticulospinal projections is offered by evidence that these responses are absent in individuated finger movements despite being present in more proximal muscles (Carlsen et al., 2008; Honeycutt et al., 2013). This may relate to the paucity of reticulospinal projections to distal muscles of the upper limb (Riddle and Baker, 2010). Finally, evidence that startReact responses are not amenable to pre-pulse inhibition, as startle responses are, may still suggest separate pathways for these responses (Valls-Solé et al., 2005, 2008), but this difference may also be explained by the higher attentional demands of task-specific preparation compared to a resting situation (Maslovat et al., 2012). The proposed model for these responses relies on the latter explanation but in doing so, describes a relatively simple system that is consistent with the sustained reliance upon giant brainstem neurons for rapid adaptive responses of a wide range of animal species.

## Conclusion

Despite being two of the most primitive and fundamental mechanisms for movement initiation, the purpose of the stretch reflex and startle response remains unclear in humans. Behavioral advantages provided by the ability to release prepared movements rapidly appear to have led to a control system in humans in which voluntary and involuntary response mechanisms overlap substantially. I suggest a number of modifications to a previous model of startle-induced movement release (startReact) involving both transcortical and subcortical pathways for sensory processing, with the transcortical pathway sharing output neurons with the transcortical stretch reflex and voluntary motor system. I suggest that transcortical pathways have evolved to provide enormous flexibility of control, complementing less flexible but faster subcortical motor pathways. Together, these motor pathways blur the boundaries between involuntary and voluntary motor control and provide us with the capacity to respond rapidly to environmental stimuli in a highly flexible and context-dependent manner.

### Conflict of interest statement

The author declares that the research was conducted in the absence of any commercial or financial relationships that could be construed as a potential conflict of interest.

## References

[B1] Ahmadi-Pajouh M. A. Towhidkhah F. Shadmehr R. (2012). Preparing to reach: selecting an adaptive long-latency feedback controller. J. Neurosci. 32, 9537–9545. 10.1523/JNEUROSCI.4275-11.2012 22787039 PMC3407880

[B2] Alibiglou L. MacKinnon C. D. (2012). The early release of planned movement by acoustic startle can be delayed by transcranial magnetic stimulation over the motor cortex. J. Physiol. 590, 919–936. 10.1113/jphysiol.2011.219592 22124142 PMC3381319

[B3] Campbell A. D. Squair J. W. Chua R. Inglis J. T. Carpenter M. G. (2013). First trial and StartReact effects induced by balance perturbations to upright stance. J. Neurophysiol. 110, 2236–2245. 10.1152/jn.00766.2012 23945786

[B4] Carlsen A. N. Chua R. Inglis J. T. Sanderson D. J. Franks I. M. (2008). Differential effects of startle on reaction time for finger and arm movements. J. Neurophysiol. 101, 306–314. 10.1152/jn.00878.2007 19005006 PMC2637008

[B5] Carlsen A. N. Maslovat D. Franks I. M. (2012). Preparation for voluntary movement in healthy and clinical populations: evidence from startle. Clin. Neurophysiol. 123, 21–33. 10.1016/j.clinph.2011.04.028 22033029

[B6] Carlsen A. N. Maslovat D. Lam M. Y. Chua R. Franks I. M. (2011). Considerations for the use of a startling acoustic stimulus in studies of motor preparation in humans. Neurosci. Biobehav. Rev. 35, 366–376. 10.1016/j.neubiorev.2010.04.009 20466020

[B7] Carter R. R. Crago P. E. Keith M. W. (1990). Stiffness regulation by reflex action in the normal human hand. J. Neurophysiol. 64, 105–118. 2388060 10.1152/jn.1990.64.1.105

[B8] Cluff T. Scott S. H. (2013). Rapid feedback responses correlate with reach adaptation and properties of novel upper limb loads. J. Neurosci. 33, 15903–15914. 10.1523/JNEUROSCI.0263-13.2013 24089496 PMC6618484

[B9] Crago P. E. Houk J. C. Hasan Z. (1976). Regulatory actions of human stretch reflex. J. Neurophysiol. 39, 925–935. 978238 10.1152/jn.1976.39.5.925

[B10] Crevecoeur F. Kurtzer I. Bourke T. Scott S. H. (2013). Feedback responses rapidly scale with the urgency to correct for external perturbations. J. Neurophysiol. 110, 1323–1332. 10.1152/jn.00216.2013 23825396

[B11] Currie S. Carlsen R. C. (1985). A rapid startle response in larval lampreys. Brain Res. 358, 367–371. 10.1016/0006-8993(85)90986-2 4075127

[B12] Curtis H. J. Cole K. S. (1942). Membrane resting and action potentials from the squid giant axon. J. Cell. Comp. Physiol. 19, 135–144 10.1002/jcp.1030190202

[B13] Davis M. Gendelman D. S. Tischler M. D. Gendelman P. M. (1982). A primary acoustic startle circuit: lesion and stimulation studies. J. Neurosci. 2, 791–805. 7086484 10.1523/JNEUROSCI.02-06-00791.1982 PMC6564345

[B14] Delval A. Dujardin K. Tard C. Devanne H. Willart S. Bourriez J.-L. (2012). Anticipatory postural adjustments during step initiation: elicitation by auditory stimulation of differing intensities. Neuroscience 219, 166–174. 10.1016/j.neuroscience.2012.05.032 22626643

[B15] Dietz V. Discher M. Trippel M. (1994). Task-dependent modulation of short-latency and long-latency electromyographic responses in upper limb muscles. Electroencephalogr. Clin. Neurophysiol. 93, 49–56. 10.1016/0168-5597(94)90091-4 7511522

[B16] Doemges F. Rack P. M. (1992). Task-dependent changes in the response of human wrist joints to mechanical disturbance. J. Physiol. 447, 575–585. 10.1113/jphysiol.1992.sp019019 1593461 PMC1176053

[B17] Eaton R. C. Bombardieri R. A. Meyer D. L. (1977). The Mauthner-initiated startle response in teleost fish. J. Exp. Biol. 66, 65–81. 870603 10.1242/jeb.66.1.65

[B18] Eaton R. C. Emberley D. S. (1991). How stimulus direction determines the trajectory of the Mauthner-initiated escape response in a teleost fish. J. Exp. Biol. 161, 469–487. 1757775 10.1242/jeb.161.1.469

[B19] Evarts E. V. Tanji J. (1974). Gating of motor cortex reflexes by prior instruction. Brain Res. 71, 479–494. 10.1016/0006-8993(74)90992-5 4219749

[B20] Fisher K. M. Zaaimi B. Baker S. N. (2012). Reticular formation responses to magnetic brain stimulation of primary motor cortex. J. Physiol. 590, 4045–4060. 10.1113/jphysiol.2011.226209 22674723 PMC3464356

[B21] Franklin D. W. Liaw G. Milner T. E. Osu R. Burdet E. Kawato M. (2007). Endpoint stiffness of the arm is directionally tuned to instability in the environment. J. Neurosci. 27, 7705–7716. 10.1523/JNEUROSCI.0968-07.2007 17634365 PMC6672883

[B22] Fullard J. H. Ratcliffe J. M. ter Hofstede H. (2007). Neural evolution in the bat-free habitat of Tahiti: partial regression in an anti-predator auditory system. Biol. Lett. 3, 26–28. 10.1098/rsbl.2006.0550 17443957 PMC2373802

[B23] Fullard J. H. Ratcliffe J. M. Soutar A. R. (2004). Extinction of the acoustic startle response in moths endemic to a bat-free habitat. J. Evol. Biol. 17, 856–861. 10.1111/j.1420-9101.2004.00722.x 15271085

[B24] Hale M. E. Long J. H. McHenry M. J. Westneat M. W. (2002). Evolution of behavior and neural control of the fast-start escape response. Evolution 56, 993–1007. 10.1111/j.0014-3820.2002.tb01411.x 12093034

[B25] Hammond P. H. (1955). Involuntary activity in biceps following the sudden application of velocity to the abducted forearm. J. Physiol. 127, P23–P25. 14354685 PMC1365709

[B26] Hammond P. H. (1956). The influence of prior instruction to the subject on an apparently involuntary neuro-muscular response. J. Physiol. 132, P17–P18. 13320395

[B27] Honeycutt C. F. Kharouta M. Perreault E. J. (2013). Evidence for reticulospinal contributions to coordinated finger movements in humans. J. Neurophysiol. 110, 1476–1483. 10.1152/jn.00866.2012 23825395 PMC4042417

[B28] Honeycutt C. F. Tresch U. A. Perreault E. J. (2015). Startling acoustic stimuli can evoke fast hand extension movements in stroke survivors. Clin. Neurophysiol. 126, 160–164. 10.1016/j.clinph.0.05.025 25002367 PMC4268121

[B29] Kearney R. E. Stein R. B. Parameswaran L. (1997). Identification of intrinsic and reflex contributions to human ankle stiffness dynamics. IEEE Trans. Biomed. Eng. 44, 493–504. 10.1109/10.581944 9151483

[B30] Kimura T. Haggard P. Gomi H. (2006). Transcranial magnetic stimulation over sensorimotor cortex disrupts anticipatory reflex gain modulation for skilled action. J. Neurosci. 26, 9272–9281. 10.1523/JNEUROSCI.3886-05.2006 16957083 PMC6674505

[B31] Koshland G. F. Hasan Z. (2000). Electromyographic responses to a mechanical perturbation applied during impending arm movements in different directions: one-joint and two-joint conditions. Exp. Brain Res. 132, 485–499. 10.1007/s002210000356 10912829

[B32] Koto M. Tanouye M. A. Ferrus A. Thomas J. B. Wyman R. J. (1981). The morphology of the cervical giant fiber neuron of Drosophila. Brain Res. 221, 213–217. 10.1016/0006-8993(81)90772-1 6793208

[B33] Krasne F. B. Edwards D. H. (2002). Modulation of the crayfish escape reflex—physiology and neuroethology. Integr. Comp. Biol. 42, 705–715. 10.1093/icb/42.4.705 21708767

[B34] Krutky M. A. Ravichandran V. J. Trumbower R. D. Perreault E. J. (2010). Interactions Between limb and environmental mechanics influence stretch reflex sensitivity in the human arm. J. Neurophysiol. 103, 429–440. 10.1152/jn.00679.2009 19906880 PMC2807231

[B35] Kurtzer I. L. Pruszynski J. A. Scott S. H. (2008). Long-latency reflexes of the human arm reflect an internal model of limb dynamics. Curr. Biol. 18, 449–453. 10.1016/j.cub.2008.02.053 18356051

[B36] Landis C. Hunt W. A. (1939). The Startle Pattern. New York, NY: Farrah and Rinebart.

[B37] Lewis G. N. Perreault E. J. MacKinnon C. D. (2005). The influence of perturbation duration and velocity on the long-latency response to stretch in the biceps muscle. Exp. Brain Res. 163, 361–369. 10.1007/s00221-004-2182-9 15654583

[B38] Liddell E. G. T. Sherrington C. S. (1924). Reflexes in response to stretch (myotatic reflexes). Proc. R. Soc. Lond. Ser. B 96, 212–242 10.1098/rspb.1924.0023

[B39] Lingenhöhl K. Friauf E. (1992). Giant neurons in the caudal pontine reticular formation receive short latency acoustic input: an intracellular recording and HRP-study in the rat. J. Comp. Neurol. 325, 473–492. 10.1002/cne.903250403 1281843

[B40] Mackie G. O. (1984). Fast pathways and escape behavior in Cnidaria, in Neural Mechanisms of Startle Behavior, ed Eaton R. C. (New York, NY: Plenum publishing), 15–42.

[B41] MacKinnon C. D. Bissig D. Chiusano J. Miller E. Rudnick L. Jager C.. (2007). Preparation of anticipatory postural adjustments prior to stepping. J. Neurophysiol. 97, 4368–4379. 10.1152/jn.01136.2006 17460098

[B42] Maslovat D. Carlsen A. N. Franks I. M. (2012). Subcortical motor circuit excitability during simple and choice reaction time. Behav. Neurosci. 126, 499–503. 10.1037/a0028285 22642891

[B43] Matthews P. B. (1991). The human stretch reflex and the motor cortex. Trends Neurosci. 14, 87–91. 10.1016/0166-2236(91)90064-2 1709536

[B44] Mitani A. Ito K. Mitani Y. McCarley R. W. (1988). Morphological and electrophysiological identification of gigantocellular tegmental field neurons with descending projections in the cat: I. Pons. J. Comp. Neurol. 268, 527–545. 10.1002/cne.902680405 3356804

[B45] Mittenthal J. E. Wine J. J. (1978). Segmental homology and variation in flexor motoneurons of the crayfish abdomen. J. Comp. Neurol. 177, 311–334. 10.1002/cne.901770209 621294

[B46] Mulloney B. (1970). Structure of the giant fibers of earthworms. Science 168, 994–996. 10.1126/science.168.3934.994 5441033

[B47] Nakazawa K. Kawashima N. Obata H. Yamanaka K. Nozaki D. Akai M. (2003). Facilitation of both stretch reflex and corticospinal pathways of the tibialis anterior muscle during standing in humans. Neurosci. Lett. 338, 53–56. 10.1016/S0304-3940(02)01353-8 12565139

[B48] Nonnekes J. Oude Nijhuis L. B. de Niet M. de Bot S. T. Pasman J. W. van de Warrenburg B. P. C.. (2014). StartReact restores reaction time in hsp: evidence for subcortical release of a motor program. J. Neurosci. 34, 275–281. 10.1523/JNEUROSCI.2948-13.2014 24381288 PMC6608175

[B49] Omrani M. Pruszynski J. A. Murnaghan C. D. Scott S. H. (2014). Perturbation-evoked responses in primary motor cortex are modulated by behavioral context. J. Neurophysiol. 10.1152/jn.00270.2014 25210158

[B50] Perreault E. J. Chen K. Trumbower R. D. Lewis G. (2008). Interactions with compliant loads alter stretch reflex gains but not intermuscular coordination. J. Neurophysiol. 99, 2101–2113. 10.1152/jn.01094.2007 18287550 PMC2810681

[B51] Preuss T. Gilly W. F. (2000). Role of prey-capture experience in the development of the escape response in the squid Loligo opalescens: a physiological correlate in an identified neuron. J. Exp. Biol. 203, 559–565. 10637184 10.1242/jeb.203.3.559

[B52] Prosser C. L. Hunter W. S. (1936). The extinction of startle responses and spinal reflexes in the white rat. Am. J. Physiol. 117, 618.

[B53] Pruszynski J. A. Kurtzer I. Nashed J. Y. Omrani M. Brouwer B. Scott S. H. (2011). Primary motor cortex underlies multi-joint integration for fast feedback control. Nature 478, 387–390. 10.1038/nature10436 21964335 PMC4974074

[B54] Pruszynski J. A. Kurtzer I. Scott S. H. (2008). Rapid motor responses are appropriately tuned to the metrics of a visuospatial task. J. Neurophysiol. 100, 224–238. 10.1152/jn.90262.2008 18463184

[B55] Ravichandran V. J. Honeycutt C. F. Shemmell J. Perreault E. J. (2013). Instruction-dependent modulation of the long-latency stretch reflex is associated with indicators of startle. Exp. Brain Res. 230, 59–69. 10.1007/s00221-013-3630-1 23811739 PMC3759548

[B56] Riddle C. N. Baker S. N. (2010). Convergence of pyramidal and medial brain stem descending pathways onto macaque cervical spinal interneurons. J. Neurophysiol. 103, 2821–2832. 10.1152/jn.00491.2009 20457863 PMC2867561

[B57] Ritzmann R. E. Camhi J. M. (1978). Excitation of Leg motor neurons by giant interneurons in the cockroachPeriplaneta americana. J. Comp. Physiol. 125, 305–316. 10.1007/BF006568653806433

[B58] Rock M. K. (1980). Functional properties of Mauthner cell in the tadpole Rana catesbeiana. J. Neurophysiol. 44, 135–150. 6968343 10.1152/jn.1980.44.1.135

[B59] Rothwell J. C. MacKinnon C. D. Valls-Sole J. (2002). Role of brainstem-spinal projections in voluntary movement. Mov. Disord. 17, S27–S29. 10.1002/mds.10054 11836749

[B60] Safavynia S. A. Ting L. H. (2013). Long-latency muscle activity reflects continuous, delayed sensorimotor feedback of task-level and not joint-level error. J. Neurophysiol. 110, 1278–1290. 10.1152/jn.00609.2012 23803325 PMC3763153

[B61] Shammah-Lagnado S. J. Negrão N. Silva B. A. Ricardo J. A. (1987). Afferent connections of the nuclei reticularis pontis oralis and caudalis: a horseradish peroxidase study in the rat. Neuroscience 20, 961–989. 10.1016/0306-4522(87)90256-9 2439943

[B62] Shemmell J. An J. H. Perreault E. J. (2009). The differential role of motor cortex in stretch reflex modulation induced by changes in environmental mechanics and verbal instruction. J. Neurosci. 29, 13255–13263. 10.1523/JNEUROSCI.0892-09.2009 19846713 PMC2791059

[B63] Sinkjaer T. Hayashi R. (1989). Regulation of wrist stiffness by the stretch reflex. J. Biomech. 22, 1133–1140. 10.1016/0021-9290(89)90215-7 2625413

[B64] Spieser L. Aubert S. Bonnard M. (2013). Involvement of SMAp in the intention-related long latency stretch reflex modulation: a TMS study. Neuroscience 246, 329–341. 10.1016/j.neuroscience.2013.05.005 23673280

[B65] Stevenson A. J. T. Chiu C. Maslovat D. Chua R. Gick B. Blouin J.-S. (2014). Cortical involvement in the StartReact effect. Neuroscience 269, 21–34. 10.1016/j.neuroscience.2014.03.041 24680855 PMC4063318

[B66] Strick P. L. (1983). The influence of motor preparation on the response of cerebellar neurons to limb displacements. J. Neurosci. 3, 2007–2020. 6619921 10.1523/JNEUROSCI.03-10-02007.1983 PMC6564559

[B67] Tanji J. Evarts E. V. (1976). Anticipatory activity of motor cortex neurons in relation to direction of an intended movement. J. Neurophysiol. 39, 1062–1068. 824409 10.1152/jn.1976.39.5.1062

[B68] Valls-Solé J. Kofler M. Kumru H. Castellote J. M. Sanegre M. T. (2005). Startle-induced reaction time shortening is not modified by prepulse inhibition. Exp. Brain Res. 165, 541–548. 10.1007/s00221-005-2332-8 15942734

[B69] Valls-Solé J. Kumru H. Kofler M. (2008). Interaction between startle and voluntary reactions in humans. Exp. Brain Res. 187, 497–507. 10.1007/s00221-008-1402-0 18458890

[B70] Valls-Solé J. Rothwell J. C. Goulart F. Cossu G. Muñoz E. (1999). Patterned ballistic movements triggered by a startle in healthy humans. J. Physiol. 516, 931–938. 10200438 10.1111/j.1469-7793.1999.0931u.x PMC2269293

[B71] Vilis T. Hore J. Meyer-Lohmann J. Brooks V. B. (1976). Dual nature of the precentral responses to limb perturbations revealed by cerebellar cooling. Brain Res. 117, 336–340. 10.1016/0006-8993(76)90743-5 825192

[B72] Wagner M. J. Smith M. A. (2008). Shared internal models for feedforward and feedback control. J. Neurosci. 28, 10663–10673. 10.1523/JNEUROSCI.5479-07.2008 18923042 PMC6671341

[B73] Yeomans J. S. Frankland P. W. (1995). The acoustic startle reflex: neurons and connections. Brain Res. Rev. 21, 301–314. 10.1016/0165-0173(96)00004-5 8806018

[B74] Yeomans J. S. Li L. Scott B. W. Frankland P. W. (2002). Tactile, acoustic and vestibular systems sum to elicit the startle reflex. Neurosci. Biobehav. Rev. 26, 1–11. 10.1016/S0149-7634(01)00057-4 11835980

